# Nitrate ammonification in mangrove soils: a hidden source of nitrite?

**DOI:** 10.3389/fmicb.2015.00166

**Published:** 2015-03-02

**Authors:** Melike Balk, Anniet M. Laverman, Joost A. Keuskamp, Hendrikus J. Laanbroek

**Affiliations:** ^1^Department of Microbial Ecology, Netherlands Institute of Ecology (NIOO–KNAW)Netherlands; ^2^Faculty of Geosciences, Utrecht UniversityUtrecht, Netherlands; ^3^CNRS EcoBio UMR6553, Université Rennes 1Rennes, France; ^4^Ecology and Biodiversity Group, Institute of Environmental Biology, Utrecht UniversityUtrecht, Netherlands

**Keywords:** nitrate reduction, ammonium production, nitrite production, mangrove soil

## Abstract

Nitrate reduction is considered to be a minor microbial pathway in the oxidation of mangrove-derived organic matter due to a limited supply of nitrate in mangrove soils. At a limited availability of this electron acceptor compared to the supply of degradable carbon, nitrate ammonification is thought to be the preferential pathway of nitrate reduction. Mangrove forest mutually differ in their productivity, which may lead to different available carbon to nitrate ratios in their soil. Hence, nitrate ammonification is expected to be of more importance in high- compared to low-productive forests. The hypothesis was tested in flow-through reactors that contain undisturbed mangrove soils from high-productive *Avicennia germinans* and *Rhizophora mangle* forests in Florida and low-productive *Avicennia marina* forests in Saudi Arabia. Nitrate was undetectable in the soils from both regions. It was assumed that a legacy of nitrate ammonification would be reflected by a higher ammonium production from these soils upon the addition of nitrate. Unexpectedly, the soils from the low-productive forests in Saudi Arabia produced considerably more ammonium than the soils from the high-productive forests in Florida. Hence, other environmental factors than productivity must govern the selection of nitrate ammonification or denitrification. A rather intriguing observation was the 1:1 production of nitrite and ammonium during the consumption of nitrate, more or less independent from sampling region, location, sampling depth, mangrove species and from the absence or presence of additional degradable carbon. This 1:1 ratio points to a coupled production of ammonium and nitrite by one group of nitrate-reducing microorganisms. Such a production of nitrite will be hidden by the presence of active nitrite-reducing microorganisms under the nitrate-limited conditions of most mangrove forest soils.

## Introduction

Mangrove forests inhabiting tropical and subtropical, coastal zones are often characterized by high primary production (Bouillon et al., 2008). Part of the primary production will be buried in the sediment leading to increased amounts of organic carbon. Decomposition of mangrove-derived carbon is essentially a process mediated by microbes (Alongi, 1988). For the oxidation of organic compounds, the microbial community has different electron acceptors at its disposal, of which oxygen is the most preferred oxidant for reasons of thermodynamics (Laanbroek, 1990). Due to diffusion limitation coupled to consumption, oxygen penetration is generally restricted in mangrove forest soils. Irrespective of inundation or air-exposure, oxygen penetrated no more than 2 mm in a mangrove forest soil (Kristensen et al., 1994). Mangrove species are also able to transfer oxygen from the air to their roots in waterlogged, anoxic soils by the presence of aerenchyma in the pneumatophores or in the prop roots, respectively (Scholander et al., 1955). Aerobic respiration and sulfate reduction are assumed to be the major pathways of mangrove-derived carbon degradation with a share of 40–50% each; see Kristensen et al. for an overview (Kristensen et al., 2008). Recent evidence suggests that the role of Fe(III) reduction in carbon oxidation may be comparable to or even higher than sulfate reduction in iron-rich mangrove environments (Kristensen et al., 2008). The contribution of nitrate reduction to mangrove-derived carbon oxidation is generally limited with a maximum observed partitioning of 19% of the benthic CO_2_ production (Kristensen et al., 2008). Low nitrate reduction rates in natural mangrove forests are likely due to inhibition of nitrifying bacteria by the lack of oxygen and concomitant low nitrate concentrations in pore waters (cf. Purvaja et al., 2008).

In general, two nitrate-reducing pathways can be recognized, i.e., (1) denitrification and (2) nitrate ammonification or dissimilatory nitrate reduction. Whereas the first pathway leads to gaseous end products (Knowles, 1982; Tiedje et al., 1982; Mahne and Tiedje, 1995), the second pathway is directed to ammonium (Ishimoto and Egami, 1959; Takahashi et al., 1963). It has been suggested that the prevalence of denitrification or nitrate ammonification is largely determined by the ratio between accessible carbon and available nitrate in the environment (Buresh and Patrick, 1978; Tiedje, 1988; Fazzolari et al., 1998; Yin et al., 2002). The nature of the residing nitrate-reducing microorganisms has also been suggested as a steering factor in the selection of the way nitrate is reduced (Brunel et al., 1992; Nijburg et al., 1998). In contrast to most of the denitrifying microorganisms that have a more oxidative metabolism, many nitrate-ammonifying bacteria have a more fermentative way of life and could therefore survive actively longer periods of oxygen and nitrate depletion.

Notwithstanding the general opinion that nitrate reduction is of limited importance due to a lack of nitrate in most mangrove soils, several studies have addressed nitrate reduction in these soils (e.g., Nedwell, 1975; Fernandes and Bharathi, 2011; Molnar et al., 2013). Denitrification rates and concomitant losses of gaseous nitrogen compounds are relatively low in mangrove soils (Rivera-Monroy et al., 1995a,b; Rivera-Monroy and Twilley, 1996; Pelegri et al., 1997; Kristensen et al., 1998; Bauza et al., 2002). Actual and potential rates of denitrification by mangrove forest soils receiving secondary sewage effluents indicated that even the nitrate-reducing communities in these nutrients-rich soils were severely nitrate limited (Corredor and Morell, 1994). Fernandes et al. (2012a,b) concluded that nitrate ammonification might be of more importance than denitrification in nitrogen-limited mangrove systems. However, the contribution of nitrate ammonification to total nitrate reduction varies largely among different mangroves soils (Giblin et al., 2013).

Primary production varies a lot among mangrove forests (Bouillon et al., 2008). Whereas healthy mangrove forests at the Atlantic coast of Florida represent highly productive forests, mangrove forests along the Red Sea coast near Jeddah, Saudi Arabia, lead a more meager existence, partly due to camel grazing. In both mangrove systems, nitrate is undetectable in the pore water of the soil. Hence, the mangrove forests from Saudi Arabia and Florida offer the possibility to study the effect of forest productivity on the ratio between nitrate ammonification and denitrification in the process of nitrate reduction. Due to the higher primary production and the likely higher carbon cycling, it was hypothesized that nitrate ammonification is relatively more important in the carbon-rich, but nitrate-limited mangrove soils from Florida than in the low-productive and carbon-poor, but also nitrate-limited soils of Saudi Arabia.

In Florida, soil samples were collected from *Avicennia germinans* and *Rhizophora mangle* sites on three different locations. Since *A. germinans* is growing in the upper, less frequently flooded and hence more oxidized parts of the intertidal zone and *R. mangle* at the lower, more often inundated and therefore more reduced parts of this zone, nitrate ammonification was expected to be more important at the sites with *R. mangle*. Due to the desert conditions at the Red Sea coast near Jeddah, *Avicennia marina* is the only species present. *A. marina* is the only well-known species that tolerates the desert climate (Macnae, 1968). Here samples were collected from different layers at two different locations, one of which was irregularly grazed by camels. Due to the more oxidized conditions at the surface of the soil, nitrate ammonification was supposed to be most important at the more reduced sub-surface layers.

Rates of nitrate reduction and of ammonium production in the mangrove forest soils were determined in flow-through reactor systems that allow for the supply of nitrate to the soil while conserving the soil structure (Roychoudhury et al., 1998; Laverman et al., 2006). Next to nitrate and ammonium, nitrite being an intermediate product of denitrification and nitrate ammonification in anoxic environments, was also determined in the outflow of the reactors.

## Materials and methods

### Sampling locations

In March 2009, soil samples were collected from monospecific stands of *Avicennia germinans* and *Rhizophora mangle* at three locations in southern Florida that differ mutually in total organic carbon content (Table 1). One sampling location was situated at the Port of the Islands (N25°54′48″ and W81°30′25″) in Collier County on the east coast of the Gulf of Mexico, one location near Jensen Beach (N27°17′00″ and W80°13′00″) in Martin County on South Hutchinson Island, and one location near Fort Pierce (N27°33′09″ and W80°19′39″) in St. Lucie County on North Hutchinson Island. North and South Hutchinson Island are part of a range of barrier islands sheltering the Indian River lagoon from the North Atlantic Ocean. Both locations are situated on the landside of the islands along the lagoon. The location at North Hutchinson Island was positioned in one of the mangrove forests impounded for mosquito control along the Indian River lagoon with limited water exchange between lagoon and impoundment through culverts (see also Verhoeven et al., 2014). Soils from *R. mangle* sites were collected from the seaward fringes, while soils from *A. germinans* were sampled more from the interior of the forests. In all cases, soils were collected from mature stands with an average tree height of more than 4 m. The pore water pH values were circumneutral with the exception of the *A. germinans* location at South Hutchinson Island, which was slightly more acidic (Table 1). The salinity of the soils was generally above ocean level with the highest salt concentrations at the *A. germinans* location at North Hutchinson Island. Concentrations of pore water nitrate and nitrite were always below detection limit (i.e., <0.5 mg/kg dry soil).

**Table 1:** **Characteristics of mangrove soil samples collected from Florida, USA, and near Jeddah, Saudi-Arabia**.

**Region**	**Location**	**Vegetation**	**Particle size of the soil (DV50)^a^**	**Total soil organic carbon (%)**	**pH**	**Salinity (g/L)**	**Nitrate (g/kg)**	**Nitrite (g/kg)**
Florida	Port of the Islands	*Avicennia germinans*	84	26	7.7	69	n.d.^b^	n.d.
		*Rhizophora mangle*	87	22	7.4	39	n.d.	n.d.
	South Hutchinson Island	*Avicennia germinans*	92	26	5.7	41	n.d.	n.d.
		*Rhizophora mangle*	116	13	7.4	37	n.d.	n.d.
	North Hutchinson Island	*Avicennia germinans*	157	3.5	7.5	95	n.d.	n.d.
		*Rhizophora mangle*	292	6.1	6.7	57	n.d.	n.d.
Saudi Arabia	Thuwal	*Avicennia marina*	73	0.7	7.7	51	n.d.	n.d.
	South Corniche	*Avicennia marina*	136	0.9	7.8	56	n.d.	n.d.

a As volume based mean diameter.

b Not detectable (<0.5 mg/kg dry soil).

In November 2009, soil samples were collected from two different *Avicennia marina* sites near the city of Jeddah, Saudi Arabia. One of the sites is located in a small, exposed zone along the coast at South Corniche (N21°16′,06″ and E39°07′30″), the other location is situated in a relatively larger mangrove area at the fringe of a sheltered creek on an island in front of the King Abdullah University for Science and Technology (KAUST) campus (N22°19′52 and E39°05′59″), which is near Thuwal, a town situated about 80 km north of Jeddah, close to northern limit of mangrove growth. The soils on both sites mainly consist of weathered corals. Irregularly grazing camels kept the plant biomass low at South Corniche. The tree height at South Corniche was always less than 1 m, while tree height at the location near Thuwal was between 1.5 and 2.0 m. Both soils were carbon-poor and pore water nitrate and nitrite concentrations were always below detection limit (Table 1).

### Sampling

Soil samples were always taken close to the roots of the trees. In Florida, replicate samples were taken within an area of 1 m^2^. In Saudi Arabia, replicate soil samples were collected at both locations approximately 1 m away from each other and parallel to the coast and creek, respectively. Soils were sampled with a specially designed shuttle corer as described by Laverman et al. (2006). The core liner of the shuttle corer consists of a stacking of reactor cells. The cells are Plexiglas rings of 2 cm height and 4.2 cm inside diameter and are enclosed in a stainless steel sleeve. The corer was hand-pushed slowly into the soil in order to avoid compaction. Samples collected in the field were stored on ice and transported to the Netherlands at 4°C. Analyses started within 1 week after sampling.

### Nitrate reduction rate determination

Nitrate reduction rates were determined with so-called flow-through reactors, which are designed to measure rates of biogeochemical reactions on undisturbed, water-saturated soils or sediments (Roychoudhury et al., 1998). A reactor contains a slice of soil within a Plexiglas ring of 2 cm height and 4.2 cm inside diameter, with 0.2 μm pore size PVDF (Millipore) and glass fiber filters (1.2 mm PALL) at each end of the cylinder. Each full reactor thus contained an undisturbed sediment slice from a well-defined depth interval. Reactors were closed with POM (poly-oxy methylene) Delrin® caps tightened with screws, with O-rings preventing leakage. Input and output channels open at the center of the caps, at the contact with the glass fiber filter. In the described analyses, the reactors contained the upper 2 cm of the sampled mangrove soils from Florida or Saudi Arabia, or the layer from 4 to 6 cm depth from Saudi Arabia.

To match the *in situ* temperature at the moment of sampling, the flow-through reactors containing the intact soil slices were placed in a thermostatic water bath to maintain the temperature throughout the incubations at 21 ± 0.5°C for the Florida samples and at 30 ± 0.5°C for the samples from Saudi Arabia, resembling the *in situ* soil temperatures at the moment of sampling. With the Florida samples, the inflow solutions contained 4 mM sodium nitrate, no electron donor and NaCl concentrations that mimicked the *in situ* salinity (Table 1). Reactors that contained the Saudi Arabia samples were all supplied with 24 mM sodium nitrate, while parallel reactors received additionally sodium acetate (10 mM) and sodium lactate (10 mM) being representative for easily degradable carbon compounds originating from diverse fermentation pathways under anoxic conditions. Reactors supplemented with nitrate but not with an exogenous carbon source are referred as non-amended.

Inflow solutions and tubing were purged with Argon before and during the incubations to maintain anoxic conditions. Inflow solutions were introduced with a peristaltic pump (a digital ISMATEC 24 channel peristaltic pump, Model 939 D) at a constant flow rate of 4.0 ± 0.1 ml h^−1^. Collection tubes were changed at indicated fixed time intervals and then stored at −18°C prior to chemical analyses. All incubations were performed in the dark to avoid oxygen production by photosynthesis. Flow-through reactor incubations were run for 90 h for the Florida samples and 40 h for the Saudi Arabia samples. The duration of the incubations was determined by the response to nitrate application. The nitrate reduction rate was calculated as follows:

$$\text{Nitrate reduction rate}=\left({\text{C}}_{\text{in}}-{\text{C}}_{\text{out}}\right)\times \text{Q/V}$$

with C_in_ is the nitrate input concentration, C_out_ is the steady-state nitrate concentration in the outflow, Q is the volumetric flow rate and V is the volume of the soil slice in the reactor (i.e., 27.7 cm^3^). Net ammonium and nitrite production rates were calculated from the ammonium and nitrite concentrations in the outflow, as no inorganic nitrogen other than nitrate was present in the inflow solution.

### Analytical methods

Inflow concentrations of nitrate and outflow concentrations of nitrate, nitrite, and ammonium were quantified by standard segmented flow colorimetric analysis on a Bran & Lübbe Autoanalyzer III. Total organic carbon measurements were conducted according to methods described in Vanbroekhoven et al. (2007). The mean particle size of the soils was measured using Sympatec HELOS BF (Germany) particle size analyzer.

### Statistical analyses

Data were analyzed in R 3.1.1 (R Core Team, 2014). Data were fitted to (mixed) linear models with treatment and location as fixed factors. While samples taken in Florida were all considered to be independent, samples from Saudi-Arabia were taken at two depths, so that a random factor was added for appropriate modeling. These data were fitted to mixed linear models using nlme 3.1-118 (Pinheiro et al., 2014). Residuals were tested for heteroscedacity using Bartlett's test and for normality using the Shapiro–Wilk test. In case of violation of either of these assumptions, this was solved through transformation of the response variable. Treatment effects were tested for significance using ANOVA with type II sum of squares using car 2.0-22 (Fox and Weisberg, 2011), while the *multcomp* 1.3-8 package (Hothorn et al., 2008) was used to test differences between the three sites in Florida using Westfall adjusted *p*-values. Adjusted *R*^2^ for mixed models were calculated using the method of Nakagawa and Schielzeth (2013).

## Results

### Nitrate consumption rates

**Figure 1:**
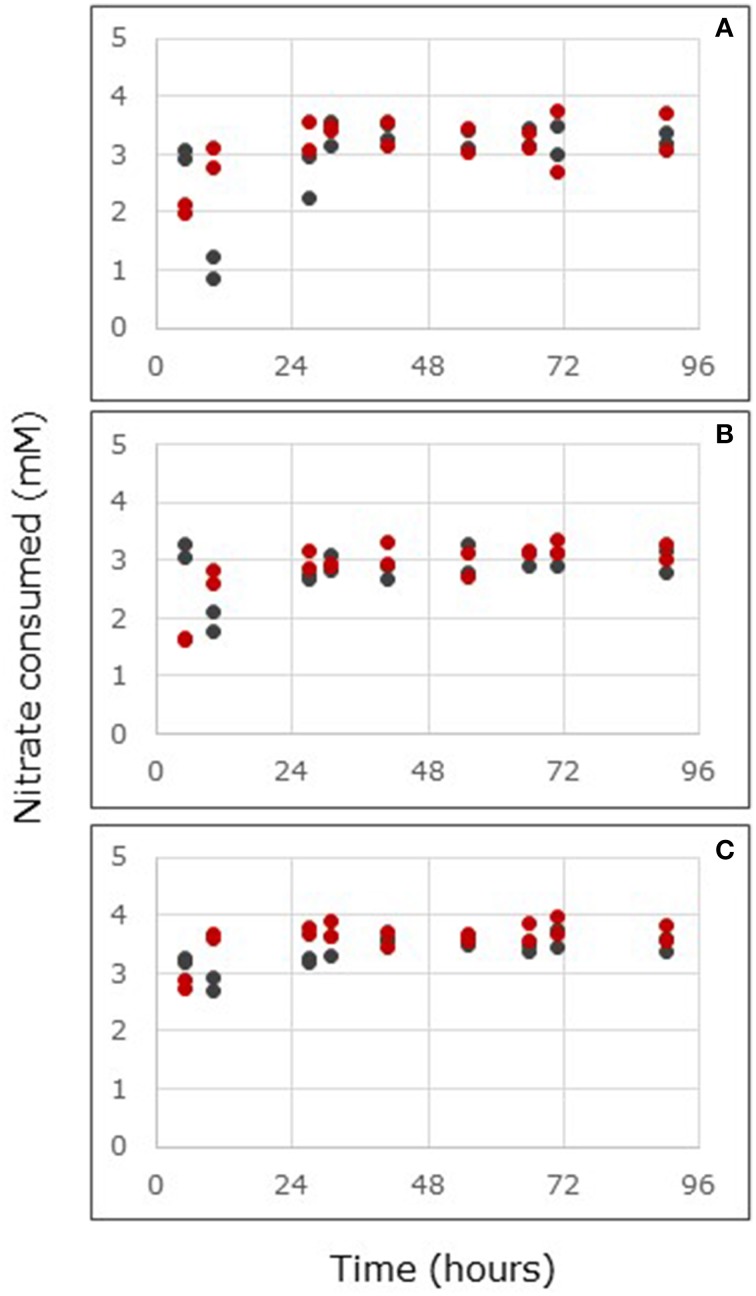
**Nitrate consumption in flow-through reactors filled with surface layers (0–2 cm depth) of mangrove soils from stands of *Avicennia germinans* (black circles) or *Rhizophora mangle* (red circles) sampled at Port of the Islands (A), South Hutchinson Island (B), and North Hutchinson Island (C), Florida.** Reactors were continuously fed with 24 mM nitrate. For each sampling location, the values of two independent reactors are presented.

**Figure 2:**
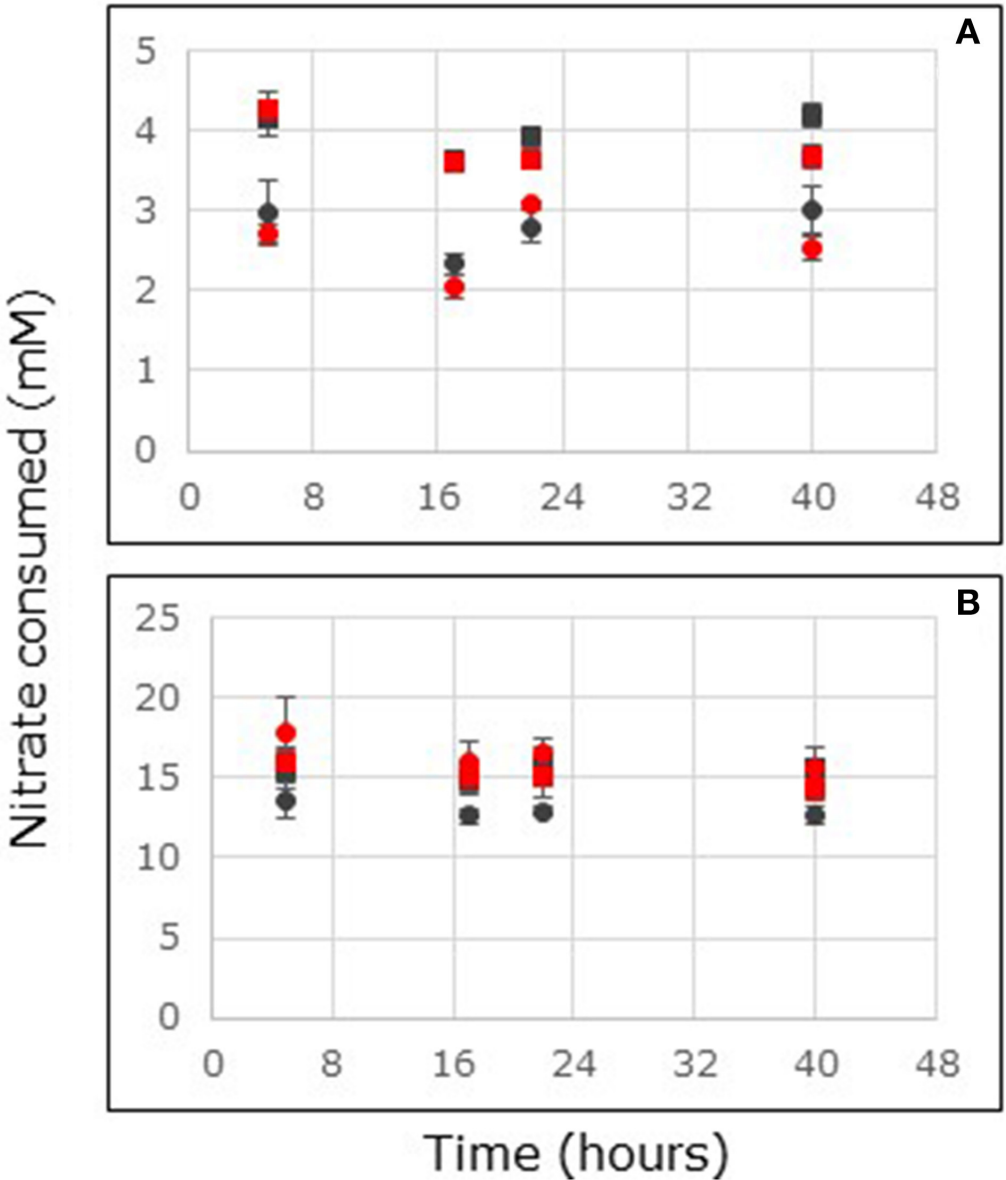
**Nitrate consumption in flow-through reactors filled with non-carbon-amended (A) and carbon-amended (B) mangrove soils collected at the surface (0–2 cm depth, circles) or sub-surface (4–6 cm depth, squares) from stands of *Avicennia marina* sampled at South Corniche (black symbols) and Thuwal (red symbols), Saudi Arabia.** Reactors were continuously fed with 24 mM nitrate. For each sampling location and depth, the average values of five independent reactors are presented (bars represent standard deviations).

Mangrove soil samples from Florida and Saudi Arabia were supplied with nitrate in the input solution of the flow-through reactors in order to determine nitrate reduction rates in the presence of excess nitrate. Nitrate concentrations in the outflow of the reactors were lower than those in the inflow solution, indicating nitrate consumption by the soils (Figures 1, 2 for the samples from Florida and Saudi Arabia, respectively). With the soil samples from Florida, steady state in nitrate consumption had been reached within 27 h. During the period after 27 h, nitrate consumption rate was significantly affected by sampling location, but not by mangrove species (Supplementary Table 1). Samples from South Hutchinson Island consumed significantly less nitrate than samples from both other locations (*p* < 0.01 for North Hutchinson Island and *p* < 0.05 Port of the Islands). Nitrate consumption was not significantly different between Port of the Islands and North Hutchinson Island.

Soils from Saudi Arabia were tested for nitrate consumption with two different soil layers i.e., 0–2 and 4–6 cm depth. Steady state nitrate consumption rates were reached within the first 17 h of the incubation (Figure 2). This held for non-carbon-amended and for carbon-amended soil samples, as well as for surface and for sub-surface samples. Carbon-amended samples showed higher nitrate consumption rates than non-carbon-amended samples. With non-carbon-amended and with carbon-amended samples, sampling depth, and sampling location affected the nitrate consumption rate significantly (Supplementary Tables 2, 3). With non-carbon-amended samples, highest rates were observed at South Corniche at 4–6 cm depth and the lowest rates at Thuwal at the surface of the soil (**Table 3**). With carbon-amended samples, the highest rates at Thuwal occurred at the surface and the lowest rates at the surface of South Corniche. A comparison between non-amended, surface samples from *A. germinans* soils in Florida and from *A. marina* soils in Saudi Arabia, demonstrated a significant (*p* < 0.001, Supplementary Table 4) effect of the sampling region on nitrate consumption with the highest values observed in the soils from Florida.

### Ammonium production rates

**Figure 3:**
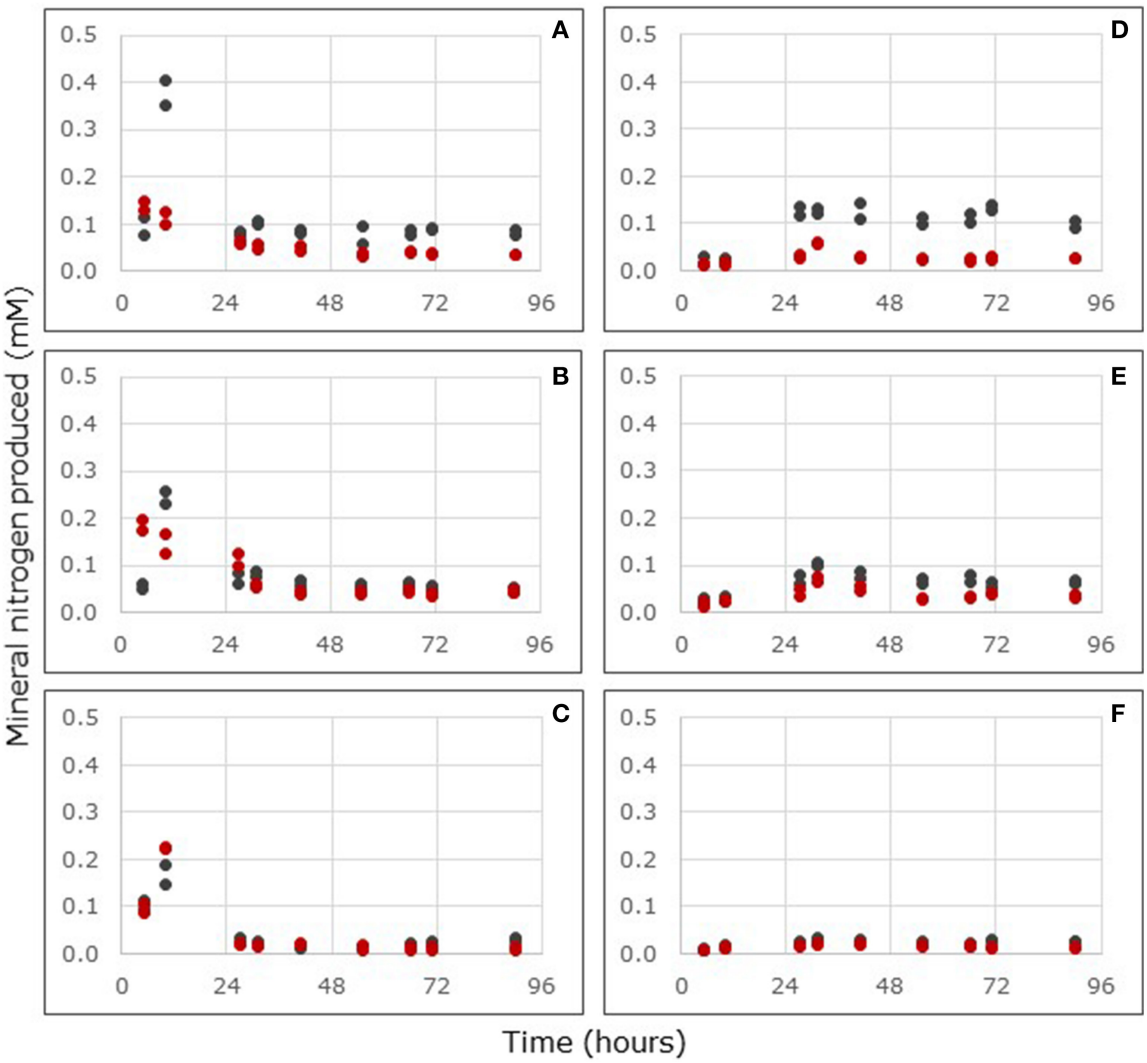
**Ammonium (A–C) and nitrite (D–F) production in flow-through reactors filled with surface layers (0–2 cm depth) of mangrove soils from stands of *Avicennia germinans* (black dots) or *Rhizophora mangle* (red dots) sampled in Port of the Islands (A,D), South Hutchinson Island (B,E), and North Hutchinson Island (C,F), Florida.** For each sampling location, the values of two independent reactors are presented.

Within 5 h after the start of the reactor incubations, soils from both Florida and Saudi Arabia started to release ammonium. With the soils from Florida, the release of ammonium showed a maximum after 10 h (Figures 3A–C). After 27 h the production of ammonium had reached steady state. During the period from 27 until 90 h, the ammonium production rate was significantly affected by mangrove species and sampling location, but a significantly interactive effect between plant species and sampling location was observed as well (Supplementary Table 1). Samples from beneath *A. germinans* produced ammonium at a significantly higher rate than samples from underneath *R. mangle* (Table 2). The ammonium production rate was significantly (*p* < 0.001) lower in samples from North Hutchinson Island than in samples from the other two locations in Florida, but the ammonium production rates in samples from Port of the Islands from South Hutchinson Island were not significantly different from each other.

**Table 2:** **Average steady state rates of nitrate reduction, ammonium production, and nitrite production observed in flow-through reactors containing the upper 2 cm of mangrove soils from different locations and mangrove vegetation in Florida**.

**Location**	**Vegetation**	**Nitrate reduction rate^a^**	**Ammonium production rate**	**Nitrite production rate**
Port of the Islands	*Avicennia germinans*	463	13 (2.8%)^b^	17 (3.7%)
	*Rhizophora mangle*	479	7 (1.5%)	5 (1.0%)
South Hutchinson Island	*Avicennia germinans*	422	9 (2.1%)	11 (2.6%)
	*Rhizophora mangle*	443	8 (1.8%)	6 (1.4%)
North Hutchinson Island	*Avicennia germinans*	502	3 (0.6%)	4 (0.8%)
	*Rhizophora mangle*	535	2 (0.4%)	3 (0.6%)

Steady state rates were averaged over the time period from 27 to 90 h after the start of the measurements.

a Rates in nmol cm^−3^ h^−1^.

b Percentage of the associated nitrate reduction rate.

**Figure 4:**
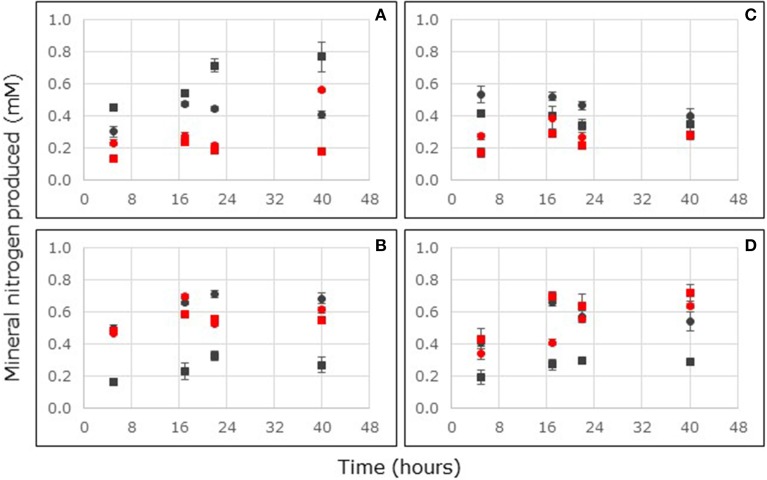
**Ammonium (A,B) and nitrite (C,D) consumption in flow-through reactors filled with non-carbon-amended (A,C) and carbon-amended (B,D) mangrove soils collected at the surface (0–2 cm depth, circles) or sub-surface (4–6 cm depth, squares) from stands of *Avicennia marina* sampled at South Corniche (black symbols) and Thuwal (blue symbols), Saudi Arabia.** Reactors were continuously fed with 24 mM nitrate. For each sampling location and depth, the average values of five independent reactors are presented (bars represent standard deviations).

Ammonium production in soils from Thuwal and South Corniche had reached steady state within 17 h after the start of the reactor incubation (Figures 4A,B). With non-carbon-amended samples, the ammonium production rate was significantly affected by sampling depth and location (Supplementary Table 2). Ammonium production rates in these non-carbon-amended samples were larger in the surface layers and at South Corniche (Table 3). With carbon-amended samples, ammonium production rates were significantly affected by sampling depth and location, but an interactive effect of depth and location was also observed (Supplementary Table 3). Ammonium production rates in these carbon-amended samples were again larger in the surface layers and at South Corniche. A comparison between non-carbon-amended, surface samples from *A. germinans* soils in Florida and from *A. marina* soils in Saudi Arabia, demonstrated significantly (*p* < 0.001, Supplementary Table 4) higher ammonium production rates in samples from Saudi Arabia.

**Table 3:** **Average steady state rates of nitrate reduction, ammonium production and nitrite production observed in flow-through reactors containing the surface (0–2 cm) or sub-surface (4–6 cm) layer of *Avicennia marina* mangrove soils from different locations in Saudi Arabia**.

**Location**	**Depth (cm)**	**Nitrate reduction rate^a^**		**Ammonium production rate**		**Nitrite production rate**	
		**Non-amended^b^**	**Carbon-amended^c^**	**Non-amended**	**Carbon-amended**	**Non-amended**	**Carbon-amended**
Thuwal	0–2	368	2313	51 (13.9%)^d^	88 (3.8%)	45 (12.2%)	77 (3.3%)
	4–6	524	2136	29 (5.5%)	81 (3.8%)	38 (7.3%)	99 (4.6%)
South Corniche	0–2	391	1839	64 (16.4%)	99 (5.4%)	67 (17.1%)	85 (4.6%)
	4–6	567	2231	35 (6.2%)	82 (3.7%)	41 (7.2%)	53 (2.4%)

Steady state rates were averaged over the time period from 17 to 40 h after the start of the measurements.

a Rates in nmol cm^−3^ h^−1^.

b Non-amended: No carbon, only 24 mM nitrate in the inflow solution.

c Carbon-amended: 10 mM acetate plus 10 mM lactate and 24 mM nitrate in the inflow solution.

d In brackets percentage of the associated nitrate reduction rate.

### Relative ammonium production rates

With non-carbon-amended soils samples, the rate of ammonium production as percentage of the rate of nitrate reduction was lowest in samples from mangrove stands from North Hutchinson Island (i.e., 0.4 and 0.6%; Table 2) and highest in samples from the surface layers from Thuwal and South Corniche (13.9 and 16.4%, respectively; Table 3). With carbon amendment, the relative ammonium production rate in Saudi Arabia samples generally decreased by a factor of 2.5. With Florida samples, mangrove species, and sampling location had both a significant effect on the relative ammonium production; again with an interactive effect of species and sampling location (Supplementary Table 1). Based on total nitrate consumption, soil samples from beneath *R. mangle* produced significantly (*p* < 0.001) less ammonium than soil samples from underneath *A. germinans* and samples from North Hutchinson Island produced significantly (*p* < 0.001) less ammonium than the other two locations that were not significantly different from each other.

With non-carbon-amended samples from Saudi Arabia, both sampling location and depth had an effect on the relative ammonium production, and again an interactive effect of sampling location and depth was observed on the relative ammonium production rate (Supplementary Table 2). The samples from South Corniche produced relatively more ammonium than the samples from Thuwal and surface samples more than the sub-surface samples. With carbon-amended samples, sampling depth and location had a significant effect on the relative ammonium production rate (Supplementary Table 3). A comparison between non-carbon-amended, surface samples from *A. germinans* soils in Florida and from *A. marina* soils in Saudi Arabia, demonstrated a significant effect of region on ammonium production rates relative to nitrate production rates (*p* < 0.001) with the highest relative rates in samples from Saudi Arabia (Supplementary Table 4).

### Nitrite production rates

Within 5 h after the start of the reactor incubations, soils from both Florida and Saudi Arabia started to release nitrite. After 27 h the production of nitrite had reached steady state in the samples from Florida (Figures 3D–F). During the period from 27 till 90 h, nitrite production rates were both significantly affected by plant species and by sampling location, with a significant interactive effect of species and location (Supplementary Table 1). Samples from underneath *A. germinans* produced significantly more nitrite than samples from beneath *R. mangle* (*p* < 0.001). Nitrite production rates were significantly lower in samples from North Hutchinson Island compared to the samples from the other sampling locations (*p* < 0.001) and samples from South Hutchinson Island produced significantly less nitrite than samples from Port of the Islands (*p* < 0.001).

Nitrite production in soils from Thuwal and South Corniche had reached steady state within 17 h after the start of the reactor incubation (Figures 4C,D). In non-carbon-amended and in carbon-amended samples, sampling depth and location affected nitrite production rates significantly (Supplementary Tables 2, 3), but an interactive effect of both depth and location was also observed. In non-carbon-amended samples, rates measured at South Corniche and at the surface were significantly higher than rates at Thuwal and at the sub-surface layer (Table 3). In carbon-amended samples, the highest and lowest rates were observed in the sub-surface layers from Thuwal and South Corniche, respectively. A comparison between non-carbon-amended, surface samples from *A. germinans* soils in Florida and from *A. marina* soils in Saudi Arabia, demonstrated significantly (*p* < 0.001) higher nitrite production rates in samples from Saudi Arabia (Supplementary Table 4).

## Discussion

### The nitrate ammonification paradox

We hypothesized nitrate ammonification to be more pronounced in high- vs. low-productive mangrove forests due to the larger input of carbon in relation to the available nitrate. However, based on our data, we should reject our hypothesis as the largest contribution of nitrate ammonification to nitrate reduction was observed in the low-productive *A. marina* forests in Saudi Arabia. Also the distribution of relative nitrate ammonification rates between mangrove species in Florida and between soil depths in Saudi Arabia was opposite to the expectations. Where we expected higher relative nitrate ammonification rates beneath *R. mangle* in Florida and in the sub-surface layers in Saudi Arabia, we observed higher relative nitrate ammonification rates underneath *A. germinans* and in samples from the surface layers from Thuwal and South Corniche. So, factors other than productivity in relation to nitrate availability and oxidative conditions (i.e., the high location of *A. germinans* in the intertidal zone compared to *R. mangle*, and the surface layer vs. the sub-surface layer) must select for nitrate ammonification or denitrification.

In the non-carbon-amended flow-through reactors, nitrate reduction is entirely dependent on the electron donors present in the sampled mangrove soils. It could be argued that differences in environmental conditions will affect the quality of the organic matter, the nature of a potential mineral electron donor as well as the traits of the decomposing, nitrate-reducing microbial community. Although the salinities in the northern part of the Red Sea belong to the highest measured in marine environments (approximately 40 PSU), the salinities measured in the Saudi Arabia soils were not larger than those observed in the soils from Florida. On the contrary, the salinities encountered in Florida soils are often larger than measured in the Saudi Arabia soils, especially so for the soils from the impounded mangrove forest at North Hutchinson Island (Table 1). Annual monthly temperatures in southern Florida range from 14–26°C in January to 24–33°C in July, while the annual monthly temperatures in Jeddah, Saudi Arabia vary from 18–29°C in January to 27–40°C in July (http://www.worldweatheronline.com/). Further, microbiological analyses may show differences in temperature sensitivity between the nitrate-reducing microorganisms from Florida and Saudi Arabia. Previously, Dong et al. (2011) observed that nitrate ammonification dominates over denitrification in tropical estuaries. Similarly, King and Nedwell (1984) and Jorgensen (1989) showed in temperate European estuarine sediments that nitrate-ammonifying microorganisms predominate over denitrifying organisms during summer periods with higher temperatures, but that the latter group predominates during autumn and winter. Recently, Kraft et al. (2014) demonstrated in coastal, sandy tidal sediments that in addition to the carbon to nitrate ratio, the supply of nitrite relative to nitrate and the microbial generation time determine whether nitrate is reduced to gaseous end products or to ammonium. Only when nitrate was added instead of nitrite at a relatively high generation time, nitrate ammonification prevailed; otherwise denitrification dominated. The authors suggested that the balance between nitrate and nitrite could be due to a complete or incomplete process of nitrification. However, it is unlikely that nitrification could have occurred in our anoxic flow-through reactors. Since the supply of nitrate was maintained at the same fixed rate in all reactors, microbes in the samples grew at the same rate during the periods of steady state consumption and production rates. Hence, differences in microbial generation times could not have caused the observed differences in nitrate reduction pathways among the samples.

### The origin of ammonium produced

Ammonium is not only produced by the process of nitrate ammonification, but could also originate from the decomposition of organic matter. In addition, the continuous flow of a mineral salt solution through the reactors may release ammonium from the soil particles. The transient accumulation of ammonium in the mangrove soils from Florida directly after the start of the reactor experiment (Figure 2) may be due to a physical release of ammonium from the soils. However, within 27 h after the start, a steady state had been reached in the production rates of ammonium. With soil organic carbon contents of 3.5 and 0.7 (Table 1), and C:N ratios of 14.77 (Laanbroek, unpublished) and 12.86 (Keuskamp et al., 2013) for the *A. germinans* soil from North Hutchinson Island and for the *A. marina* soil from Thuwal, respectively, the soil organic nitrogen contents of the North Hutchinson Island and Thuwal soils amounted to 0.24 and 0.05%, respectively. When assuming that all ammonium produced in the non-amended reactors originated from nitrogen mineralization, the Thuwal soil with an ammonium production rate of 51 nmol N cm^−3^ h^−1^ (Table 3) would have mineralized its soil organic nitrogen pool 81 times faster than the North Hutchinson Island soil with an ammonium production rate of 3 nmol N cm^−3^ h^−1^ (Table 2). Such a difference in assumed organic nitrogen decomposition between the two mangrove soils is rather unlikely, which suggests that part of the ammonium in the soils from Saudi Arabia must have originated from nitrate ammonification.

### Unexpected nitrite production

**Figure 5:**
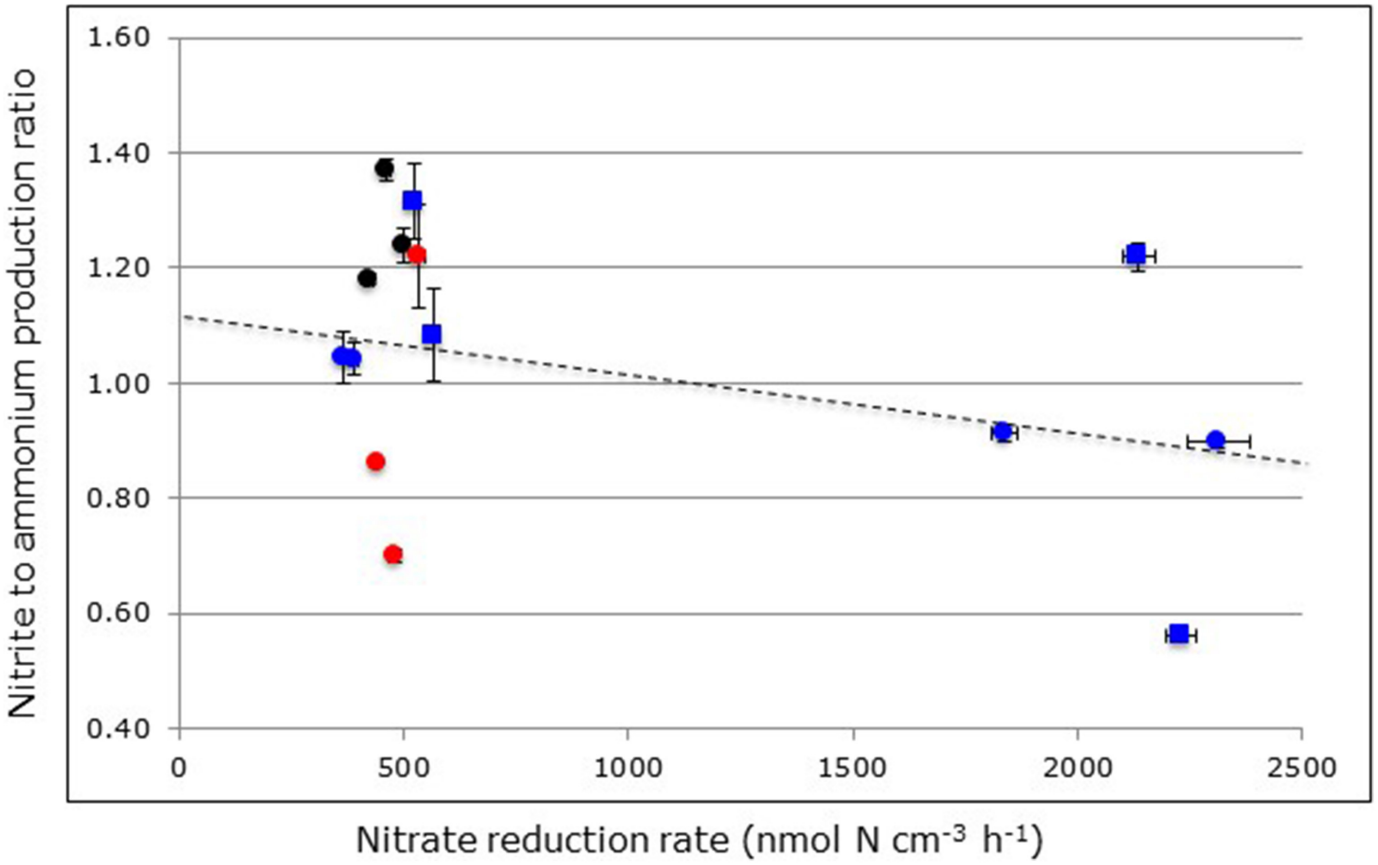
**Relationship between nitrite to ammonium production ratios and nitrate reduction rates measured in through-flow reactors filled with mangrove soils collected from *Avicennia germinans* (black circles) and *Rhizophora mangle* (red circles) mangrove forests in Florida or filled with surface (blue circles) and sub-surface (blue squares) soils from *Avicennia marina* mangrove forests in Saudi Arabia.** Reactors were continuously fed with excess nitrate (24 mM). Symbols at the left-hand side of the figure represent non-carbon-amended soils and symbols at the right-hand side carbon-amended soils. The broken line indicates the linear regression between nitrite to ammonium production ratios and the nitrate reduction rate with *R*^2^_adj_ = 0.0182 and *p* < 0.0063.

In contrast to ammonium, nitrite can only originate from nitrate reduction in the anoxic reactors. Notwithstanding the large range of nitrite production rates in relation to nitrate reduction rates (i.e., from 0.6% in the *R. mangle* soils from North Hutchinson Island to 16.4% in the surface soils from South Corniche, Tables 2, 3), the nitrite to ammonium ratios were remarkably similar in the different soil samples (Figure 5). The largest ratio deviating positively from 1.00 was observed in reactors filled with *A. germinans* soils from Port of the Islands (average ratio 1.49) and the largest ratio deviating negatively from 1.00 in reactors filled with carbon-amended, sub-surface soils from South Corniche (average ratio 0.56). In the reactors filled with soils from Florida, both sampling location and plant species affected the nitrite to ammonium production ratio significantly (Supplementary Table 1), whereas also an interactive effect of both sampling depth and location was observed. In both non-carbon-amended and carbon-amended soils from Saudi Arabia, sampling locations and sampling depth had a significant effect on the nitrite to ammonium production ratio (Supplementary Tables 2, 3).

## Concluding remarks

During our study on nitrate reduction characteristics in mangrove forest that differ clearly in productivity, we observed that:

- Contrary to our expectation, the production of ammonium from nitrate was most pronounced in the soil samples least productive mangrove forests. A good explanation is missing, but might be found in the presence of different nitrate-reducing communities due to divergent environmental conditions. Hence, a study based on relative numbers of genes specific for either the nitrate ammonification or denitrification pathway may shed light on this phenomenon.
- Nitrite and ammonium were produced from nitrate in an almost 1:1 ratio at all locations, depths and plant species studied, and independently on the absence or presence of added, easily degradable carbon. Such a constant ratio makes it rather improbable that nitrite and ammonium production are performed by two different groups of nitrate-reducing microorganisms. It seems much more likely that both oxidized nitrogen compounds are the product of one metabolic way. To our knowledge, no nitrate-ammonifying bacterium has been isolated that is known to couple the production of ammonium directly to the production of nitrite. Recently, Streminska et al. (2012) showed that nitrate-ammonifying bacteria isolated from a sandy soil with high nitrate concentration, produced ammonium and nitrite simultaneously during anaerobic growth on nitrate. However, the nitrite to ammonium ratio was dependent on the bacterial species studied and on the nature of the electron donor, and the production ratio was rather different from the 1:1 ratio that we observed in mangrove soils. Isolation of the responsible nitrate-ammonifying bacteria from the mangrove soils and subsequent physiological studies may shed light on the mechanism behind this ratio. In a nitrate-limited environment such as mangrove forest soils, such a concomitant production of nitrite and ammonium by the cells will remain hidden as nitrite will immediately be used as an alternative electron acceptor for the oxidation of carbon.

### Conflict of interest statement

The authors declare that the research was conducted in the absence of any commercial or financial relationships that could be construed as a potential conflict of interest.
