# Supplementary material for: Nitrate ammonification in mangrove soils: a hidden source of nitrite?
Source: Front Microbiol. 2015 Mar 2;6:166. doi: 10.3389/fmicb.2015.00166 (PMC4345912; doi:10.3389/fmicb.2015.00166)
Supplement: Supplementary file 1 [file Table1.DOCX]

**Supplementary Table 1**⏐ ANOVA tables for steady state nitrogen conversion rates measured in nitrate-saturated flow-through reactors filled with surface layers from stands of *Avicennia germinans* and *Rhizophora mangle* collected from Port of the Islands, South Hutchinson Island and North Hutchinson Island, Florida.

| **Dependent variables** | **Independent variable** | **Sum of squares** | **Df** | **F value** | **p (>F)** | |
| --- | --- | --- | --- | --- | --- | --- |
| Nitrate reduction rate | Species | 0.346 | 1 | 1.930 | 0.214134 |  |
|  | Location | 7.186 | 2 | 20.030 | 0.002211 | ** |
|  | Location : Species | 0.199 | 2 | 0.555 | 0.601101 |  |
|  | Residuals | 1.076 | 6 |  |  |  |
| Ammonium production rate | Species | 29.203 | 1 | 41.382 | 0.0006672 | *** |
|  | Location | 89.456 | 2 | 63.380 | 9.231e-05 | *** |
|  | Species : Location | 16.211 | 2 | 11.486 | 0.0088830 | ** |
|  | Residuals | 4.234 | 6 |  |  |  |
| Relative ammonium production rate | Species | 0.000143 | 1 | 56.025 | 0.0002938 | *** |
|  | Location | 0.000450 | 2 | 87.631 | 3.627e-05 | *** |
|  | Species : Location | 0.000066 | 2 | 12.912 | 0.0066943 | ** |
|  | Residuals | 0.000015 | 6 |  |  |  |
| Nitrite production rate | Species | 113.849 | 1 | 116.800 | 3.714e-05 | *** |
|  | Location | 104.517 | 2 | 53.613 | 0.0001488 | *** |
|  | Species : Location | 71.106 | 2 | 36.475 | 0.0004389 | *** |
|  | Residuals | 5.848 | 6 |  |  |  |
| Nitrite to ammonium production ratio | Species | 0.34762 | 1 | 130.836 | 2.679e-05 | *** |
|  | Location | 0.11682 | 2 | 21.985 | 0.0017312 | ** |
|  | Species : Location | 0.30835 | 2 | 58.027 | 0.0001188 | *** |
|  | Residuals | 0.01594 | 6 |  |  |  |

Significance codes: *** 0.001, ** 0.01, * 0.05
