# Supplementary material for: Nitrate ammonification in mangrove soils: a hidden source of nitrite?
Source: Front Microbiol. 2015 Mar 2;6:166. doi: 10.3389/fmicb.2015.00166 (PMC4345912; doi:10.3389/fmicb.2015.00166)
Supplement: Supplementary file 2 [file Table2.DOCX]

**Supplementary Table 2**⏐ ANOVA tables for steady state nitrogen conversion rates measured in nitrate-saturated, non-carbon-amended flow-through reactors filled with surface (0 – 2 cm deep) layers and sub-surface (4 – 6 cm deep) layers from stands of *Avicennia marina* collected from South Corniche and Thuwal, Saudi Arabia.

| **Dependent variables** | **Independent variable** | **Chi^2^** | **Df** | **p (>Chi^2^)** | |
| --- | --- | --- | --- | --- | --- |
| Nitrate reduction rate | Depth | 1113.868 | 1 | <2.2e-16 | *** |
|  | Location | 17.197 | 1 | 3.37e-05 | *** |
|  | Depth : Location | 3.739 | 1 | 0.05317 |  |
| Ammonium production rate | Depth | 549.049 | 1 | <2.2e-16 | *** |
|  | Location | 65.453 | 1 | 5.953e-16 | *** |
|  | Depth : Location | 1.545 | 1 | 0.2139 |  |
| Relative ammonium production rate | Depth | 1032.233 | 1 | <2.2E-16 | *** |
|  | Location | 29.605 | 1 | 5.297e-08 | *** |
|  | Depth : Location | 3.764 | 1 | 0.05236 |  |
| Nitrite production rate | Depth | 252.157 | 1 | <2.2e-16 | *** |
|  | Location | 140.178 | 1 | <2.2e-16 | *** |
|  | Depth : Location | 77.376 | 1 | <2.2e-16 | *** |
| Nitrite to ammonium production ratio | Depth | 20.946 | 1 | 4.725e-06 | *** |
|  | Location | 0.706 | 1 | 0.4007 |  |
|  | Depth : Location | 19.560 | 1 | 9.748e-06 | *** |

Significance codes: *** 0.001, ** 0.01, * 0.05
