# Supplementary material for: Nitrate ammonification in mangrove soils: a hidden source of nitrite?
Source: Front Microbiol. 2015 Mar 2;6:166. doi: 10.3389/fmicb.2015.00166 (PMC4345912; doi:10.3389/fmicb.2015.00166)
Supplement: Supplementary file 3 [file Table3.DOCX]

**Supplementary Table 3**⏐ ANOVA tables for steady state nitrogen conversion rates measured in nitrate-saturated, carbon-amended flow-through reactors filled with surface (0 – 2 cm deep) layers and sub-surface (4 – 6 cm deep) layers from stands of *Avicennia marina* collected from South Corniche and Thuwal, Saudi Arabia.

| **Dependent variables** | **Independent variable** | **Chi^2^** | **Df** | **p (>Chi^2^)** | |
| --- | --- | --- | --- | --- | --- |
| Nitrate reduction rate | Depth | 6.904 | 1 | 0.008602 | ** |
|  | Location | 15.883 | 1 | 6.739e-05 | *** |
|  | Depth : Location | 47.915 | 1 | 4.450e-12 | *** |
| Ammonium production rate^1^ | Depth | 13.838 | 1 | 0.0001993 | *** |
|  | Location | 248.170 | 1 | <2.2e-16 | *** |
|  | Depth : Location | 17.703 | 1 | 2.582e-05 | *** |
| Relative ammonium production rate^1^ | Depth | 27.767 | 1 | 1.368e-07 | *** |
|  | Location | 141.499 | 1 | <2.2e-16 | *** |
|  | Depth : Location | 28.877 | 1 | 7.713e-08 | *** |
| Nitrite production rate | Depth | 21.888 | 1 | 2.891e-06 | *** |
|  | Location | 197.780 | 1 | <2.2e-16 | *** |
|  | Depth : Location | 553.705 | 1 | <2.2e-16 | *** |
| Nitrite to ammonium production ratio | Depth | 5.260 | 1 | 0.02182 | * |
|  | Location | 569.852 | 1 | <2.2e-16 | *** |
|  | Depth : Location | 595.981 | 1 | <2.2e-16 | *** |

Significance codes: *** 0.001, ** 0.01, * 0.05

^1^ Without carbon-amended, sub-surface samples from South Corniche (residuals not normally distributed)
