# Supplementary material for: Nitrate ammonification in mangrove soils: a hidden source of nitrite?
Source: Front Microbiol. 2015 Mar 2;6:166. doi: 10.3389/fmicb.2015.00166 (PMC4345912; doi:10.3389/fmicb.2015.00166)
Supplement: Supplementary file 4 [file Table4.DOCX]

**Supplementary Table 4**⏐ ANOVA tables for steady state nitrogen conversion rates measured in nitrate-saturated, non-carbon-amended flow-through reactors filled with surface (0 – 2 cm deep) layers from stands of *Avicennia germinans* and *Avicennia marina* collected from the regions Florida and Saudi Arabia, respectively.

| **Dependent variables** | **Independent variable** | **Sum of squares** | **Df** | **F value** | **p (>Chi^2^)** | |
| --- | --- | --- | --- | --- | --- | --- |
| Nitrate reduction rate | Region | 31276 | 1 | 35.746 | 3.376e-05 | *** |
|  | Residuals | 12249 | 14 |  |  |  |
| Ammonium production rate | Region | 9292.7 | 1 | 245.36 | 2.866e-10 | *** |
|  | Residuals | 530.2 | 14 |  |  |  |
| Relative ammonium production rate | Region | 0.068035 | 1 | 378.68 | 1.558e-11 | *** |
|  | Species | 0.002515 | 14 |  |  |  |
| Nitrite production rate | Region | 7920.6 | 2 | 80.217 | 3.593e-07 | *** |
|  | Residuals | 1382.4 | 14 |  |  |  |
| Nitrite to ammonium production ratio | Region | 0.27455 | 1 | 29.301 | 9.144e-05 | *** |
|  | Residuals | 0.13118 | 14 |  |  |  |

Significance codes: *** 0.001, ** 0.01, * 0.05
